# A Systematic Review of the Effect of Polyphenols on Alterations of the Intestinal Microbiota and Shared Bacterial Profiles Between Metabolic Syndrome and Acne

**DOI:** 10.3390/nu16213591

**Published:** 2024-10-23

**Authors:** Sara Ilari, Saverio Nucera, Lucrezia Morabito, Rosamaria Caminiti, Valeria Mazza, Giovanna Ritorto, Sara Ussia, Lucia Carmela Passacatini, Roberta Macrì, Federica Scarano, Maria Serra, Elisabetta Scali, Jessica Maiuolo, Francesca Oppedisano, Ernesto Palma, Saverio Muscoli, Stefania Proietti, Carlo Tomino, Vincenzo Mollace, Carolina Muscoli

**Affiliations:** 1 IRCCS San Raffaele Roma, 00166 Rome, Italy; carmela.passacatini@sanraffaele.it (L.C.P.); carlo.tomino@sanraffaele.it (C.T.); 2 Department of Health Sciences, Institute of Research for Food Safety and Health (IRC-FSH), University “Magna Graecia” of Catanzaro, 88100 Catanzaro, Italy; saverio.nucera@hotmail.it (S.N.); lucreziamorabito13@gmail.com (L.M.); rosamariacaminiti4@gmail.com (R.C.); valeria.mazza001@studenti.unicz.it (V.M.); giovanna.ritorto@studenti.unicz.it (G.R.); saraussia1598@gmail.com (S.U.); robertamacri85@gmail.com (R.M.); federicascar87@gmail.com (F.S.); maria.serra@studenti.unicz.it (M.S.); scali@unicz.it (E.S.); maiuolo@unicz.it (J.M.); oppedisanof@libero.it (F.O.); palma@unicz.it (E.P.); mollace@libero.it (V.M.); 3 Department of Cardiology, Tor Vergata University, 00133 Rome, Italy; saveriomuscoli@gmail.com; 4 Agea, Coordination Body, 00185 Rome, Italy; s.proietti@agea.gov.it

**Keywords:** nutraceuticals, microbiota, bacteria, metabolism, systematic review

## Abstract

**Introduction:** Microbiota, composed of micro-organisms like bacteria, viruses, and non-pathogenic fungi, plays a crucial role in digestion, vitamin production, and protection against dangerous microbes. Several factors, including age, diet, alcohol consumption, stress, environmental microorganisms, and therapies (particularly antibiotics), as well as birth and nursing, could modify the microbiota. Recent research has highlighted its alteration and involvement in a various disease, including metabolic syndrome and acne. This systematic review aimed to identify common biomarkers and microbiota alterations shared between metabolic syndrome and acne, and to explore how the potential prebiotic activities of polyphenols may promote intestinal eubiosis. **Materials and methods:** A comprehensive search in PubMed and EMBASE resulted in 4142 articles, from which nine studies were selected based on specific criteria after removing duplicates and reviewing abstracts and full texts. All studies correlated the microbiota alteration in both pathologies and the activity of polyphenols in metabolic syndrome. **Results:** This review suggests that acne may be influenced by some of the same microorganisms involved in metabolic syndrome. While the literature highlights the effectiveness of polyphenols in treating metabolic syndrome, no studies have yet demonstrated their specific impact on acne. **Conclusions:** The research points to the potential benefits of polyphenols in modulating the microbiota, which could be relevant for individuals with metabolic syndrome. However, due to the limited data available, it was not possible to establish a direct correlation between metabolic syndrome and acne.

## 1. Introduction

### 1.1. Gut Microbiota

**Figure 1:**
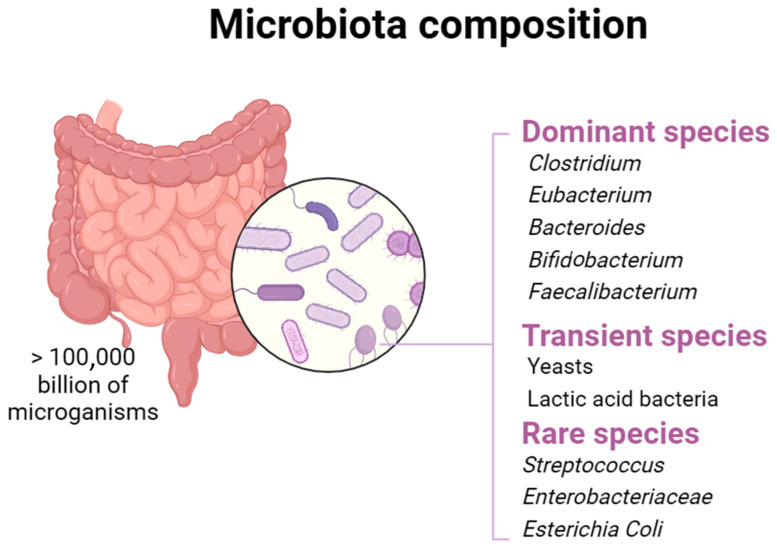
Different microbial species inhabit the host’s gut microbiota.

The gut microbiota, also known as the gut flora, is a community of microorganisms that reside in the human gastrointestinal tract. This community consist of a large number of cells, estimated at up to 10^13^, that belong to all three domains of life (*Bacteria*, *Archaea*, and *Eukaryotes*) [1]. The gut can host thousands of bacterial species belonging to different phyla, including *Bacteroidetes*, *Firmicutes*, *Actinobacteria*, *Proteobacteria*, *Verrucomicrobia*, *Fusobacteria*, *Tenericutes*, *Spirochaetes*, *Cyanobacteria*, and *Saccharibacteria* [2,3]. In contrast, the diversity of fungi in the human gut is limited. From samples of 98 human adults, a pyrosequencing study identified about 66 fungal genera, with *Saccharomyces*, *Candida*, and *Cladosporium* being the most prevalent [1,4]. The gut microbiota plays a pivotal role in maintaining host health, influencing various physiological functions and contributing to digestion, nutrient absorption, immune system maturation, and protection against harmful pathogens [5]. A healthy balance of the gut microbiota is often associated with good health, while an imbalance, known as “dysbiosis”, can be linked to various pathological conditions [6]. The gut microbiota plays a crucial role in the fermentation of non-digestible substrates such as dietary fiber and endogenous intestinal mucosa. This activity favors the development of specialized microorganisms that generate short-chain fatty acids (SCFAs) such as acetate, propionate, and butyric acid, and gas [7]. Butyric acid, which represents the main energy source for colon cells, positively impacts human health. This substance can stimulate the intestine’s production of glucose and encourage the colon’s natural death of cancer cells, which helps control blood sugar levels and preserve a healthy energy balance [8]. In addition, butyrate is essential in facilitating the consumption of high oxygen amounts by epithelial cells through β-oxidation. This activity generates a state of hypoxia, which helps to preserve the oxygen intestinal balance and prevent any imbalances in the microbiota. Propionate is transported to the liver, regulating gluconeogenesis and satiety through interaction with intestinal fatty acid receptors [9]. Acetate, the most present among SCFAs, represents an essential metabolite for bacteria proliferation, spreading into peripheral tissues and participating in cholesterol metabolism and lipogenesis [10]. Foods contribute significantly to the intestinal microbiota composition, suggesting that nutrition can prevent or alleviate diseases related to an altered microbial composition. As previously mentioned, the intestinal microbiota is involved in the food substrates fermentation, promoting or inhibiting the growth of specific types of bacteria [11]. Beyond nutrition, probiotics currently represent the most effective and safe method to specifically modify the intestinal microbiota and promote the improvement of the host’s health (Figure 1).

### 1.2. Intestinal Microbiome in Adults with Acne Vulgaris

In the 1930s, Stokes and Pillsbury used experimental data to establish a connection between inflammation of the skin and microbial flora. They noted that up to 40% of people with acne showed hypochlorhydria, suggesting that hydrochloric acid deficiency could induce migration of bacteria from the colon tract to the small intestine, disturbing the balance of normal intestinal flora [12]. In recent years, it has been confirmed that hypochlorhydria represents a significant risk factor for bacterial overgrowth in the small intestine (SIBO), which can cause an increase in intestinal permeability (“leaky gut”), resulting in the development of systemic inflammation [13,14].

*Bifidobacteria* and *Lactobacilli*, normally present in the intestine as lactic-acid-producing bacteria, could play a beneficial role in the treatment of inflammatory skin diseases, including acne [15].

The gut microbiome plays a key role in the management of both systemic and local inflammation through interaction with the immune system. Microbial communities support the integrity of the intestinal barrier by converting complex, indigestible polysaccharides into short-chain fatty acids and vitamins, especially K and B12. The integrity of the intestinal barrier, supported by mucus, immune cells, IgA, and antimicrobial peptides (AMPs) produced by epithelial cells, is fundamental in preventing the access of intestinal bacteria to the bloodstream, thus contributing to the maintenance of skin homeostasis [1].

A study highlighted a significant decrease in the presence of *Actinobacteria*, *Bifidobacterium*, *Butyricicoccus*, *Coprobacillus*, and some *Lactobacillus* species in people suffering from acne vulgaris, accompanied by a significant increase in *Proteobacteria* [16,17].

Furthermore, studies have observed that acne patients have a low presence of *Firmicutes* and an increase of *Bacteroidetes*. According to one hypothesis, the stimulation of sterol regulatory element-binding protein-1 (SREBP-1), fatty acids, and sebum triglycerides occurs following an interruption of the nutritional signal, favoring the proliferation of *Propionibacterium acnes* [17]. Several metabolic pathways are involved in the onset of acne vulgaris, such as the mTOR pathway which is activated by high blood sugar levels [18]. This event contributes to increasing insulin-like growth factor signaling, improving the cytoplasmic expression of FoxO1, ultimately leading to the development of acne [19].

### 1.3. Intestinal Microbiota in Adults with Metabolic Syndrome

**Figure 2:**
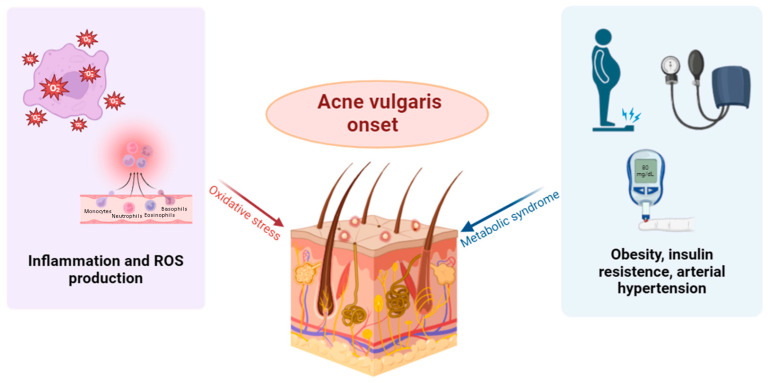
Oxidative stress and metabolic syndrome contribute to the development of acne vulgaris.

In recent years, several studies have suggested that gut microbiota dysbiosis is closely associated with many metabolic diseases, including obesity, diabetes, and non-alcoholic fatty liver disease (NAFLD). A reduction in the abundance of *Akkermansia muciniphila*, *Faecalibacterium prausnitzii*, and *Bacteroides* is observed, contrasted with a significant increase in the *Phylum Firmicutes* [20]. A metagenome analysis of a cohort of lean and obese Chinese adolescents showed a significant decrease in *Bacteroides thetaiotaomicron*, with a negative correlation in serum glutamate concentration [21]. Animal studies confirmed that the administration of *B. thetaiotaomicron* reduced serum concentrations in branched-chain amino acids and mitigated diet-induced weight gain and obesity in mice [22,23]. The gut microbiota can ferment indigestible carbohydrates, producing important metabolites such as SCFAs and succinate. These metabolites play a significant role in obesity and its comorbidities. SCFAs regulate energy balance and prevent obesity by suppressing appetite and increasing energy expenditure [24]. Several studies found significant differences in the composition of the gut microbiota between patients with type 1 diabetes (T1DM) and healthy people [25]. A study performed on Swedish participants highlighted a significant alteration of the intestinal microbiota in patients with type 2 diabetes (T2DM) [26], compared to healthy people. In fact, in the intestines of T2DM patients, a decrease in the amount of *Bifidobacteria* and *Akkermansia* and an increase in *Dallella* were observed [27]. Hypertension and atherosclerosis contribute to the cardiovascular risk associated with metabolic syndrome. In people with hypertension, a significant decrease in the richness, diversity, and uniformity of the intestinal microbiota is observed [28], together with a crucial increase in the *Firmicutes*/*Bacteroidetes* ratio. Other research has indicated a possible connection between intestinal microbiota (GM) and cardiovascular diseases (CVD), underlining bacterial translocation from the intestine to the heart [6,29]. The presence of live bacteria and bacterial DNA detected in atherosclerotic plaques suggests the implication of the microbiome in the development and progression of atherosclerosis. In atherosclerotic patients, the abundance of *Enterobacteriaceae* and *Enterobacter aerogenes* was significantly higher than in control samples, with an inhibitory effect on the growth of beneficial bacteria [30]. At the same time, several studies, both on animal and human models, have highlighted that intestinal dysbiosis associated with atherosclerosis could increase intestinal permeability, thus favoring the absorption of lipopolysaccharides in circulation [31]. Pathophysiological mechanisms associated with metabolic syndrome, intestinal dysbiosis, and acne vulgaris include chronic inflammation and elevated levels of oxidative stress, which may mediate the connection between the two disorders [32] (Figure 2). These data underline a close correlation between metabolic syndrome and intestinal microbiota, suggesting that the microbiota equilibrium could represent a promising therapeutic strategy to manage this condition.

### 1.4. The Role of Polyphenols in Gut Microbiota Modulation

Polyphenols are natural phytochemical compounds present in many plants, fruits, and herbal remedies, including green tea, black tea, apples, coffee, bergamot, and grapes. To date, approximately 8000 different types of polyphenols have been identified [33]. Based on their chemical structure, polyphenols can be roughly divided into several categories, including condensed tannins, hydrolyzable tannins, phlorotannins, polyphenolic caffeoyl ester derivatives, flavones and flavonoids, and other substances. These polyphenolic compounds perform various beneficial activities, including antioxidant action [28,34], anti-inflammatory properties [35], and antibacterial activity [36]. Polyphenols show remarkable potential in improving several health conditions, including cancer, Alzheimer’s disease [37], diabetes, NAFLD, insulin resistance, inflammatory bowel disease, and arthritis [38,39,40,41,42,43,44]. Polyphenols offer different opportunities for interaction with the intestinal microbiota, mainly due to their wide presence in foods, herbal medicines, and plants, and thus in our diet [43,45,46]. Polyphenols are characterized by an important range of effects, thanks to different molecular structures which create multiple possible interaction with the intestinal microbiota. The low bioavailability of many monomeric polyphenols, as demonstrated by Wan and colleagues in 2021, means that a significant proportion of polyphenol can reach the gastrointestinal tract without being fully absorbed. Finally, they are used directly by microorganisms [47]. The interaction between intestinal microbiota and polyphenols occurs mainly in three distinct ways:

(1) Polyphenols influence the composition of the intestinal microbiota; (2) they alter the metabolic processes within the microbial system; (3) the intestinal microbiota itself is involved in the processing of polyphenols, influencing their structure and activity. This complex interaction plays a critical role in modulating the biological effect of polyphenols within the intestinal environment, offering greater insight into how these compounds influence host health [33].

**Figure 3:**
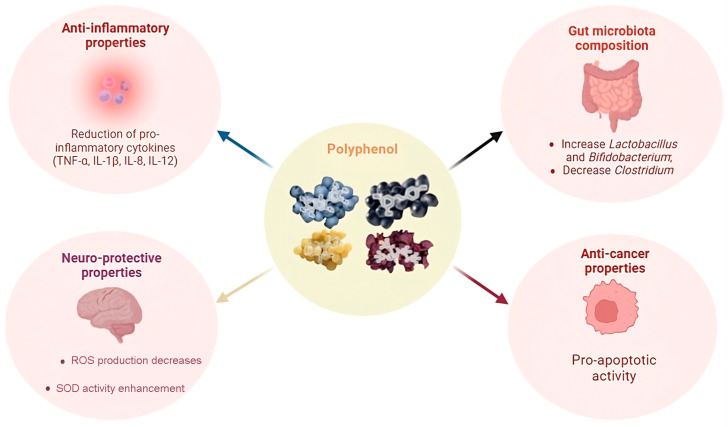
Properties of polyphenols.

Polyphenols are present in different sources such as *Smilax china* L. extracts, along with tea polyphenols and those of *Aronia melanocarpa* (*Michx.*). They have demonstrated the ability to positively influence the diversity of microbial species in the intestine, known as α-diversity. This influence has been observed in contexts such as metabolic disorders caused by a high-fat diet (HFD). Other polyphenols, including litchi pulp phenols, kaempferol, and resveratrol, have been associated with improving the α-diversity of the gut microbiota, contributing to the relief of colitis. Subsequently, polyphenols were detected as agents capable of altering the overall structure of the intestinal microbiota, evidenced by variations in β-diversity. These structural changes have been observed in individuals suffering from various pathologies, including obesity, hepatic steatosis, atherosclerosis, and colitis. Data analysis revealed a difference in the structure of the gut microbiota between sick and healthy people. Such structural changes have also been found in individuals treated with polyphenols. In conclusion, the action of polyphenols in modulating both α-diversity and β-diversity highlights their significant role in promoting intestinal health and contributing to the treatment of microbiota-related disorders [33]. A study conducted by Lu et al. in 2016 highlighted that polyphenols deriving from green tea have an important role in acne management [48]. A significant decrease in inflammatory lesions was noted on specific areas of the adults’ skin undergoing treatment, thanks to the anti-inflammatory and antioxidant action of these compounds (Figure 3).

## 2. Objectives

This systematic review aims to identify common biomarkers and microbiota alterations that may indicate significant links between metabolic syndrome and acne. Additionally, it explores how the prebiotic properties of polyphenols could contribute to maintaining intestinal eubiosis, providing a strong scientific rationale for their potential use in therapeutic and preventive approaches.

## 3. Materials and Methods

### 3.1. Search Strategy

The systematic review integrates data from experimental studies regarding the changes in the human intestinal microbiome in adults affected by acne and/or metabolic syndrome, exploring the polyphenols’ effect in improving these conditions. The keywords used to search for the articles were: “(acne) AND (gut microbiota)”, “(polyphenols) AND (acne)”, “(Lactobacillus) AND (acne) AND (Proteobacteria)”, “(polyphenols) AND (microbiome) AND (metabolic syndrome)”, “(phenols) AND (gut microbiome)”, “(gut microbiota) AND (metabolic syndrome)”.

### 3.2. Information Sources

The studies included in the review were retrieved from the PubMed and EMBASE databases, following the PRISMA 2020 Checklist (Preferred Reporting Items for Systematic Reviews and Meta-Analyses) statement and the PICO (Patient, Intervention, Comparison, Outcome) framework [49]. All articles written in English were evaluated, and the search did not specify a specific time frame. An evaluation of references of all retrieved articles was also conducted to identify additional relevant studies not included in the aforementioned databases.

### 3.3. Eligibility Criteria

Studies of the following types were included in the analysis:

- studies in women and men with acne;
- studies in women and men with metabolic syndrome or diseases associated with this condition;
- studies reporting oral polyphenol treatments.

Studies were excluded from the analysis for the following reasons:

- use of topical treatments.
- studies conducted on animals;
- studies conducted in vitro.

### 3.4. Study Results

Two separate qualitative analyses were conducted to account for the heterogeneity of the results. In the first systematic analysis, the aim was to identify the composition variations of the gut microbiota in adults with acne, comparing different clinical studies conducted on men and women. The second systematic analysis compared different human experimental models to better understand variations in gut microbiota composition in people with metabolic syndrome or related conditions. This second analysis examined the effect of oral polyphenol-based treatment on the gut microbiota and showed a qualitative improvement.

### 3.5. Statistical Analysis

Due to the bacteria and data heterogeneity and the limited number of specific studies on the chosen topic, it was necessary to analyze the data at a qualitative level.

## 4. Results

### 4.1. Study Selection

**Figure 4:**
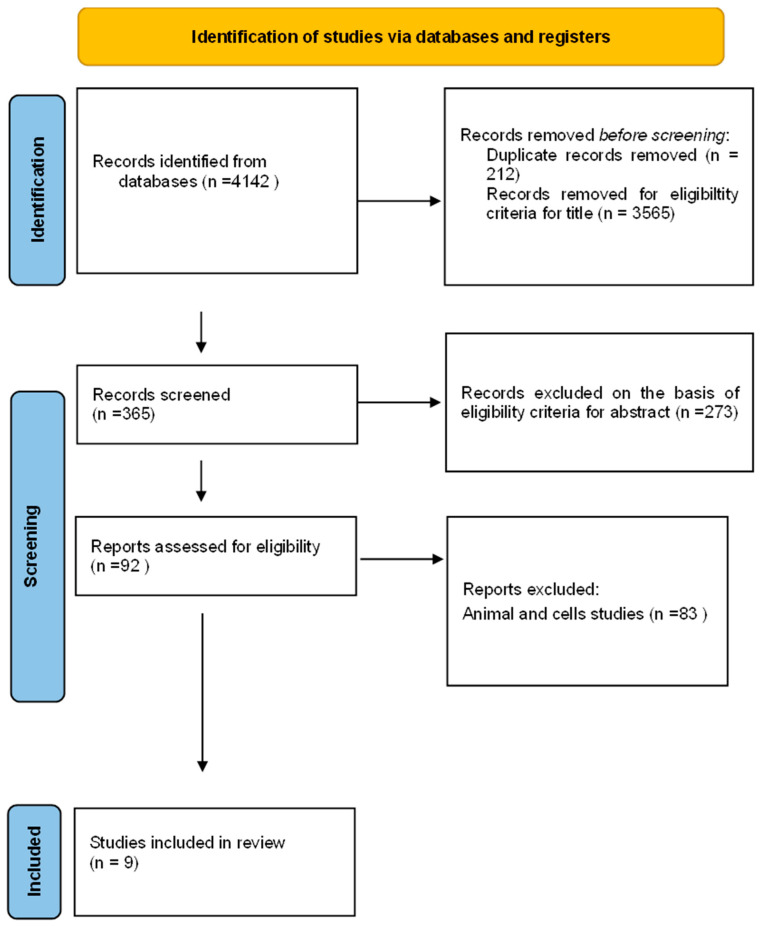
PRISMA flowchart.

The systematic review identified 4142 documents from the literature search. Of these, 212 articles were excluded as duplicates, and 3565 were excluded as their content did not match the review’s objectives. After screening the abstract and full text, 365 articles were assessed for their eligibility, and 88 were excluded because they were reviews or animal studies, involved topical administrations, or had incomplete data. Finally, nine articles describing studies on humans were included in the qualitative analysis (systematic review) and divided into two analyses, one relating to in vivo studies on patients with acne and one relating to in vivo studies on patients with metabolic syndrome. The literature search process and the selection of articles are illustrated in the following Figure (PRISMA flowchart Figure 4).

### 4.2. Study Characteristics

#### 4.2.1. Systematic Review on Gut Microbiome Changes in Acne Patients

Table 1 summarizes the main features of the four articles describing the differences in gut microbiome composition between healthy and acne patients. Formerly, it has been observed that gut microbiota plays a crucial role in the pathogenesis of acne. Hui-Min Yan et al. (2018) conducted a study to identify the differences in gut microbiota between acne patients and healthy individuals [28]. The study enrolled 31 healthy individuals (free from any skin disease) and 31 individuals with moderate to severe acne vulgaris. The distribution of the phyla *Proteobacteria* and *Actinobacteria*, the two major populations in the human gut, shows a significant disparity between acne patients and controls [28]. The phylum *Proteobacteria* is more abundant in acne patients, including many pathogenic bacteria such as *E. coli*, *Salmonella*, and *Vibrio cholerae* [28]. In addition, a decreased level of the genera *Bifidobacterium*, *Butyricicoccus*, *Coprobacillus*, *Lactobacillus*, and *Allobaculum* is observed in acne patients [28]. *Bifidobacterium* are Gram-positive bacilli belonging to the phylum *Actinobacteria*, while *Lactobacillus* are Gram-positive bacilli belonging to the phylum *Firmicutes*. A subsequent analysis conducted by Katherine G. Thompson et al. (2020) was published based on a case-control study investigating the skin and gut microbiota in eight acne patients before and after oral minocycline administration, comparing them with eight controls [50]. Oral antibiotics are the “gold standard” for the treatment of moderate to severe acne. So far, it is not known whether the efficacy of oral antibiotics in acne treatment is due to systemic absorption and, therefore, to the direct action of the antibiotic on the acne lesion, or an indirect action, due to the action of the antibiotic on the gut microbiome. Compared to controls, acne patients showed a reduced gut ratio of *Firmicutes* to *Bacteroidetes*. Following oral antibiotic treatment, acne patients’ microbiome underwent a statistically significant increase in *Bacteroidetes* levels. Furthermore, the gut microbiota in acne patients at baseline, compared to controls, was observed to be poor in Gram-positive probiotic bacilli such as *Lactobacillus iners*, *Lactobacillus zeae*, and *Bifidobacterium animalis* [50]. It has also been observed that increased intestinal permeability may allow the passage of lipopolysaccharides (LPS) by Gram-negative gut bacteria into systemic circulation, promoting inflammation and the development of acne lesions through their action on toll-like receptors TLR2 and TLR4, which are overexpressed in inflammatory acne [50]. Deng and colleagues studied whether the gut microbiota is altered in individuals with acne. It was shown that differences in microbial diversity were found between patients with acne and controls. At the phylum level, the *Firmicutes abundance* was lower in the patient group compared to healthy controls, while that of *Bacteroidetes* was higher [51]. The study conducted by Manzhalii et al. examined the effect of *Escherichia coli* Nissle on intestinal inflammation and the intestinal microbiota in patients with intestinal dermatoses. After treatment with *E. coli* Nissle, a significant reduction in pathogenic flora and an increase in *Bifidobacteria* and *Lactobacilli* were observed in the feces of treated patients. This change in the microbiota was associated with a clinical improvement, with normalization of stool consistency, color, and odor. Additionally, an increase in serum IgA levels and suppression of proinflammatory cytokines were found, indicating a beneficial effect of probiotic therapy with *E. coli* Nissle in intestinal dermatoses [52].

#### 4.2.2. Systematic Review on Changes in the Gut Microbiome in Adults with Metabolic Syndrome or Associated Pathological Conditions

Table 2 presents an overview of the articles considered in the systematic review, which aim to describe the changes in the gut microbiome in adults with metabolic syndrome or pathological conditions closely related to it. In addition, the improvements in the microbiome following the administration of substances/supplements/diets/drinks with a high polyphenolic content are described. In the study carried out by Moreno-Indias et al. in 2016, the possible prebiotic effect deriving from moderate consumption of red wine polyphenols on the modulation of the composition of the gut microbiota and the improvement of risk factors associated with metabolic syndrome in obese patients was evaluated [53]. In the study, 20 patients were involved, 10 of whom were healthy adults. An increased level of *Proteobacteria* and *Firmicutes* has been demonstrated in patients with metabolic syndrome compared to healthy adults. However, after the red wine and de-alcoholized red wine intake, no significant differences at the phylum level emerged between the two study groups [53]. Adults with metabolic syndrome, after the periods of treatment based on red wine polyphenols, presented a higher concentration of *Fusobacteria* and *Bacteroidetes* and a significant decrease in *Firmicutes*, compared to baseline [54]. Within *Firmicutes*, in patients with metabolic syndrome, a lower number of bacteria from the *Clostridium* group and the *Clostridium histolyticum* group was found, accompanied by a significant increase in bacteria belonging to the *Blautia coccoides–Eubacterium rectale*, *Faecalibacterium prausnitzii*, *Roseburia*, and *Lactobacillus* groups, after periods of red wine and de-alcoholized red wine intake compared to baseline. Finally, it was observed that the increase in *Actinobacteria* and *Lactobacillus*, together with the decrease in *Clostridium histolyticum* and *E. coli*, predicted the triglycerides’ reduction. Instead, the decrease in plasma LPS levels was associated with the *Bifidobacterium* growth and the reduction in *Enterobacter cloacae* number [53]. The study conducted by Vaiserman et al. in 2017 on the Ukrainian population demonstrates the changes in the composition of the gut microbiota between a group of “normal weight” individuals and a group of patients classified as “obese” based on body mass index (BMI). The study found a significant increase in *Firmicutes* and a higher *Firmicutes*/*Bacteroidetes* ratio in overweight and obese people compared to normal-weight adults in the Ukrainian population. The results obtained in the Ukrainian population are consistent with those obtained in other populations [54].

The traditional Chinese herbal formula Yangyin Tiluo Decoction (YTD), rich in polyphenols and used in the study by Ni et al. in 2017, mitigates metabolic syndrome and cardiovascular diseases [55]. In this study, the gut microbiota alterations in elderly people with metabolic syndrome, following natural treatment based on polyphenols, were investigated. In these patients, YTD can improve intestinal microbial composition and reduce the metabolic markers of cerebrovascular disease [55]. Older adults with metabolic syndrome showed an increase in *Lactobacillus* and *Bifidobacterium* species, accompanied by a reduction in butyrate-producing bacteria compared to healthy controls. Although *Lactobacillus* and *Bifidobacterium* are generally considered beneficial bacteria and often used as probiotics, studies have shown an increase in interleukin 1β (IL-1β) concentrations and a potential increase in the inflammatory response following their presence. YTD can reduce the lipoprotein A concentration, a known risk factor for cardiovascular disease and atherosclerosis. The decrease in lipoprotein A reflects the clinical efficacy of YTD in mitigating metabolic syndrome in older individuals [55].

A correct diet is essential to maintain the balance of the intestinal bacterial flora. According to Meir et al. (2021), a “green” diet rich in vegetable-derived polyphenols brings benefits to individuals affected by hepatic steatosis, abdominal obesity, and dyslipidemia [56]. The hypothesis has been advanced that the intestinal microbiota plays a crucial role in the pathogenesis of NAFLD, as dysbiosis appears to be linked to the modulation of fat and lipid hepatic metabolism. In this study, nine bacteria at the genus level were initially identified as significantly related to IHF (intrahepatic fat); these represented 5% of bacteria at the genus level, including *Fournierella*, *Anaerosporobacter*, *Lachnospiraceae_UCG-003*, and several genera of the *Ruminococcaceae* family [56]. Out of the nine bacteria identified, eight of them showed an association with the variation in IHF 18 months from the beginning of the study, suggesting that the presence of these bacteria is linked to changes in intrahepatic fat over time. Therefore, adopting a “green” diet enriched in polyphenols combined with physical exercise has been shown to significantly reduce the prevalence of NAFLD and improve intestinal bacterial flora [56]. Finally, in a randomized crossover clinical trial performed by Zhang et al., 2022, it is evaluated whether supplementation with polyphenol-rich red fruits (RRB), alone or in association with a prebiotic (FOS), improves cardiovascular risk biomarkers in adults with prediabetes and insulin resistance, and whether these effects are related to the modulation of the intestinal microbiota [57]. Supplementation with RRB has been shown to reduce hepatic insulin resistance, total cholesterol (TC), and LDL cholesterol (LDL-C) levels in the group of individuals with prediabetes and insulin resistance [57]. Supplementation with RRB increased the presence of *E. eligens* and reduced that of *R. gnavus*, while supplementation with RRB + FOS increased the *Bifidobacterium* spp. presence and decreased that of *B. wexlerae*. Variations in the abundances of *R. gnavus*, *E. eligens*, and *B. catenulatum* were significantly correlated with a reduction in hepatic insulin resistance and a decrease in TC and LDL-C [57]. *R. gnavus* is a fundamental component of the gut microbiota, characterized by the ability to degrade mucus [58]. Evidence suggests an association of *R. gnavus* with several pathologies, including diverticular disease, respiratory allergies in children, and septic arthritis. Furthermore, it is significantly more present in patients with Crohn’s disease, indicating inflammatory dysbiosis of the intestine [59]. The presence of 300% of *R. gnavus* was observed in the PreDM-IR group compared to the control group, which was decreased following supplementation with RRB, a result like that observed in the previously cited study on the Mediterranean “green” diet [60]. Overall, RRB supplementation improved hepatic insulin resistance, total cholesterol, and LDL cholesterol levels, with these effects being correlated with decreased *R. gnavus* and increased *E. eligens* [57]. The addition of FOS to the RRB regimen further enhanced β-cell function and increased the presence of *Bifidobacterium* spp., a gut bacterium known for its beneficial effect [57].

## 5. Discussion

Acne, in addition to being a common skin condition, plays an indicative role in detecting alterations in the gut microbiome, similar to what occurs in metabolic syndrome.

The intestinal microbiome plays a fundamental role in maintaining the host’s health, exerting a significant impact on several physiological processes, including digestion, nutrient absorption, maturation of the immune system, and defense against harmful pathogens.

An optimal balance of the microbiota is closely related to an optimal state of health, while an imbalance, known as “dysbiosis”, can be associated with various pathological conditions and diseases; systemic and/or local inflammation is a consequence of SIBO which leads to an increase in intestinal permeability and the passage of bacteria into the systemic circulation [14].

Bacterial dysbiosis in adults with acne mainly concerns an increased ratio of *Firmicutes*/*Bacteroidetes* and *Proteobacteria*; one notable study reported the presence of *Actnobacteria*, *Bifidobacterium*, *Butyricicoccus*, *Coprobacillus*, and some species of *Lactobacillus*, positive bacteria for the balance of the intestinal bacterial flora [16].

Literature studies show a specific imbalance in gut flora composition in people with metabolic syndrome. This alteration is characterized by an increase in the *Firmicutes*/*Bacteroidetes* ratio and in *Bifidobacteria* reduction; similar conditions can be found in individuals suffering from acne [61,62].

In this systematic review, although it was not possible to obtain a quantitative relevance of intestinal bacteria in adults with acne due to the limited amount of data available, an appreciable qualitative difference emerged. In patients suffering from acne compared to the control group, a significant increase in the presence of bacteria belonging to the *Phylum Proteobacteria* including pathogenic bacteria such as *E. coli*, *Salmonella*, and *Vibrio cholera* and an increased *Firmicutes*/*Bacteroidetes* ratio have been observed. On the other hand, lower levels of the genera *Bifidobacterium* (*Phylum Actinobacteria*), *Butyricicoccus*, *Coprobacillus*, *Lactobacillus* (*Phylum Firmicutes*), and *Allobaculum* are found in acne patients. It has been highlighted that, at the basal level, the microbiota of acne patients, when compared to controls, showed a significant deficiency of Gram-positive probiotic bacilli such as *Lactobacillus iners*, *Lactobacillus zeae*, and *Bifidobacterium animalis* [63].

Systematic analysis of the bacteria in the gut flora of people with metabolic syndrome shows similarities to changes in the microbiome of people with acne. The microbiomes of adults with metabolic syndrome or closely related pathologies have higher levels of *Proteobacteria* and *Firmicutes* than healthy controls. The *Firmicutes*/*Bacteroides* ratio is also found to be increased in people with obesity.

Treatment with polyphenols leads to an improvement in the balance of the intestinal bacterial flora, which correlates with a significant improvement in several cardiometabolic parameters [56]. These improvements include reductions in total cholesterol, triglycerides, and LDL, as well as a significant incidence of hepatic steatosis.

The bacterial flora restoration induced by polyphenols is characterized by a greater concentration of *Fusobacteria* and *Bacteroidetes*, accompanied by a notable reduction of the *Firmicutes* compared to the controls. Within the *Firmicutes phylum*, a reduced number of bacteria belonging to the *Clostridium* and *Clostridium histolyticum* groups was found, while a significant increase in bacteria was observed in the *Blautia coccoides–Eubacterium rectale*, *Faecalibacterium prausnitzii*, *Roseburia*, and *Lactobacillus* groups. In addition, the polyphenol treatment stimulated the increase of *Bifidobacterium* spp., an intestinal bacterium known for its beneficial effects. This review suggests that acne is caused by some of the same microorganisms that alter in metabolic syndrome. The literature only demonstrates the effectiveness of polyphenols in metabolic syndrome, with no study reporting this effect in acne. This research suggests that the potential benefits of polyphenols at the microbiota level may be relevant for patients suffering from metabolic syndrome. However, due to a lack of data, a direct correlation between metabolic syndrome and acne cannot be established.

### Limitations of the Study

Some data observed could influence the results regarding the efficacy of polyphenols in metabolic syndrome or acne. Natural compounds come from different families, increasing heterogeneity. A further critical issue is represented by the bacteria heterogeneity, which prevented conducting the meta-analysis. The limited quantity of items represents a further difficulty. Current information on the potential positive effect of polyphenols on acne is limited; however, it is known that they exert a beneficial effect on the intestinal microbiota. The positive role of polyphenols in reducing cardiovascular risk also emerges from the qualitative analysis carried out on metabolic syndrome. Another important limitation is that anthropometric parameters, such as age, medication use, and the consumption of antibiotics, probiotics, and prebiotics, which can significantly influence the composition of the gut microbiota, were not taken into account.

## 6. Conclusions

The role of the intestinal microbiome in the development of diseases is unequivocal, even though the results currently available are limited. The gut microbiota acts decisively in the pathological processes that lead to various chronic conditions. In this context, polyphenols derived from plant species seems to hold great potential in the management and prevention of disease. The positive interaction between polyphenols and microbiota opens the way to new strategies for the therapeutic management of chronic diseases and the development of personalized therapies aimed at improving patient recovery during rehabilitation. It is therefore necessary to highlight how implementing a balanced diet, with a high content of polyphenols, can bring important enhancement to the microbiota. Furthermore, considering the beneficial effects of polyphenols on the bacteria involved in metabolic syndrome, this approach could provide new foundations for their introduction in patients with acne, as the bacteria involved are the same. Therefore, a new holistic approach should include both the use of polyphenols and a balanced diet for improved health.

## Figures and Tables

**Table 1:** Studies highlighting changes in the gut microbiota in adults affected by acne.

Study/Year	Population	Age	Sex	Bacteria Phylum	Results
Hui-Min Yan et al., 2019 [28]	31 healthy adults; 31 adults with acne	Healthy adults: 22.87 ± 3.65 Acne: 22.16 ± 4.24	Male 8 Female 26	*Actinobacteria* *Proteobacteria*	It has been demonstrated that the phylum *Actinobacteria* is lower in patients with acne compared to the control group, while *Proteobacteria* is higher compared to the control group.
Yongqiong Deng et al., 2018 [51]	43 healthy adults; 43 adults with acne	Healthy adults: 20.24 ± 3.06 Acne: 20.19 ± 2.59	Male 17 Female 26	*Bacteroidetes* *Firmicutes*	In the study, differences in microbial diversity were found for patients with acne and controls. At the phylum level, the *Firmicutes* abundance was lower in the patient group compared to healthy controls, but that of *Bacteroidetes* was higher.
Katherine G. Thompson et al., 2020 [50]	8 healthy adults; 8 adults with acne	Healthy adults: 23~34. Acne: 20~32	NA	*Firmicutes* *Bacteroidetes*	It has been observed that at the phylum level, the abundance of *Firmicutes* was lower in the patient group compared to healthy controls, but that of *Bacteroidetes* was higher.
Elina Manzhalii et al., 2016 [52]	20 healthy adults; 37 adults with acne	Healthy adults: 28 ± 2.5 Acne: 29 ± 3.1		*Actinobacteri* (*Genus Bifidobcteria*) *Firmicutres* (*Genus Lactobacteria*)	In treatment with *E. coli* Nissle, a significant reduction in pathogenic flora and an increase in *Bifidobacteria* and *Lactobacilli* were observed in the feces of treated patients.

**Table 2:** Studies highlighting changes in the gut microbiome in adults with metabolic syndrome or related conditions.

Study/Year	Population	Age	Sex	Bacteria Phylum	Polyphemols	Results
Moreno-Indias et al., 2016 [53]	10 healthy adults; 10 adults with metabolic syndrome	48 ± 2 years (range 45–50 years)	NA	*Fusobacteriota* *Bacteroidetes* *Firmicudes*	Red wine polyphenols	Significant increase in *Fusobacteria* and *Bacteroidetes*, and significant decrease in the *Clostridium* and *Firmicudes* group, in patients with metabolic syndrome, compared to baseline.
Vaiserman et al., 2017 [54]	34 adults with BMI ≤18.5–24; 11 adults with BMI >30	Mean age 44.2 years	Adults with BMI ≤18.5–24: 9 male and 25 female; adults with BMI >30: 4 male and 7 female	*Actinobacteria* *Firmicutes* *Bacteroidetes*	NA	Adults with a high BMI have a higher level of *Firmicutes* and a lower level of *Bacteroidetes* than adults with normal weight.
Ni et al., 2018 [55]	11 healthy adults; 12 adults with metabolic syndrome	60–89 years	Heathy people: 6 male and 5 female; metabolic syndrome: 8 male and 4 female	*Firmicutes* *Bacteroidetes* *Actinobacteria* *Proteobacteria*	Yangyin Tiluo Decoction (YTD)	Adults treated with the Chinese herbal formula (Yangyin Tiluo Decoction) were able to reduce the abundance of potentially pathogenic bacteria and lipoprotein A.
Meir et al., 2021 [56]	294 adults with NAFLD adulted to a different diet: 98 MMED 98HFG 98 green MED	51 years	260 male and 34 female	*Firmicutes*	A polyphenol-rich diet (440–800 mg/day)	Adults treated with a diet rich in polyphenols (green MED) demonstrated a reduction in non-alcoholic hepatic steatosis, beneficial changes in cardiometabolic and inflammatory parameters, and improvements in intestinal bacterial flora.
Zhang et al., 2022 [57]	10 healthy adults; 26 with pre-diabetes	35 ± 2 years	Heathy adults: 3 male and 7 female; pre-diabetes adults: 14 male and 12 female	*Actinobacteria* *Firmicutes*	Red berry polyphenols	Supplementation with red fruit polyphenols (RRB) reduced total cholesterol and LDL levels in adults with pre-diabetes; RRB increased *Eubacterium eligens* and reduced *Ruminococcus gnavus*, while RRB + FOS increased *Bifidobacterium* spp. and reduced *Blautia wexlerae* compared to baseline.

## Data Availability

Not applicable.
